# Eugenol Reduces LDL Cholesterol and Hepatic Steatosis in Hypercholesterolemic Rats by Modulating TRPV1 Receptor

**DOI:** 10.1038/s41598-019-50352-4

**Published:** 2019-09-30

**Authors:** Amani A. Harb, Yasser K. Bustanji, Ihab M. Almasri, Shtaywy S. Abdalla

**Affiliations:** 1 Department of Biological Sciences, School of Science, The University of Jordan, Amman, 11942 Jordan; 2 Department of Biopharmaceuticals and Clinical Pharmacy, School of Pharmacy, The University of Jordan, Amman, 11942 Jordan; 3 Hamdi Mango Center for Scientific research, The University of Jordan, Amman, 11942 Jordan; 4 Department of Pharmaceutical Chemistry and Pharmacognosy, Faculty of Pharmacy, Al-Azhar University, Gaza, Palestine

**Keywords:** Receptor pharmacology, Obesity

## Abstract

Eugenol, a component of essential oils of medicinal and food plants, has a hypolipidemic effect in experimental animals although its mechanism of action is still unclear. This study aims to explore the mechanism of the hypolipidemic effect of eugenol in rats fed a high cholesterol and fat diet (HCFD). Eugenol significantly reduced total cholesterol (TC), low-density lipoproteins (LDL), atherogenic index (AI) but not high-density lipoproteins (HDL) or triglycerides (TG). Eugenol also decreased steatosis and hepatic inflammation in liver sections, decreased hepatomegaly, and the hepatic marker enzymes alanine aminotransferase (ALT) and alkaline phosphatase (ALP) activity and increased the antioxidant enzymes superoxide dismutase (SOD) and catalase (CAT) activity in hypercholesterolemic rats. Eugenol did not inhibit hepatic 3-hydroxy-3-methyl-glutaryl-CoA (HMG-CoA) reductase but caused down-regulation of transient receptor potential vanilloid (TRPV1) channels in the liver. Docking simulation using fast, rigid exhaustive docking (FRED) software indicated a tail-up/head-down interaction of eugenol with TRPV1 channel. Data indicate that eugenol does not inhibit HMG-CoA reductase but rather induces its action by interaction with TRPV1 channels.

## Introduction

Eugenol (4-allyl-2-methoxyphenol) is a major component of the essential oil of clove (*Eugenia caryophyllus*) and other medicinal and aromatic plants^1,2^. It has been considered as a safe nutrient, with the acceptable daily intake of up to 2.5 mg/kg body weight for humans, as recommended by the Joint FAO/WHO Expert Committee on Food Additives^3^. Eugenol is found as a functional ingredient of many products used in pharmaceutical, food and cosmetic industry^4^. So it was used as a food supplement and as therapeutic ingredient in several medications such as many dental preparations and as an enhancer of skin permeation of diverse drugs. Eugenol even found agricultural applications, since it was used as a pesticide and fumigant and to protect foods from microorganisms during storage^4,5^.

Numerous biological activities of eugenol have been documented including antioxidant^1,6,7^, anti-inflammatory^1,6,8^, anti-aging^7^, and anticancer activities^1^. Eugenol also reduces the risk of developing cardiovascular diseases such as hypertension, hyperlipidemia and diabetes. The vasodilatory, hypotensive, bradycardic, and hypoglycemic effects of eugenol were also reported^2,8–10^.

The role of eugenol in lipid metabolism has been demonstrated in several laboratories. Parts of many medicinal plants that contain eugenol have hypolipidemic activity including the essential oil of leaves of *Ocimum minimum* L.^11^, the essential oil of leaves of *Melissa officinalis* L. (lemon balm)^12^ and cloves^13^. Recently, animal studies have shown that eugenol lowers serum cholesterol levels and inhibits lipogenesis in the liver, hence strongly suggesting that eugenol may protect against atherosclerosis and fatty liver disease^5,14–16^. Furthermore, aspirin eugenol ester was synthesized and tested on hyperlipidemic rats. It reduced TC, TG and LDL effectively and it was more effective than its individual components (aspirin, eugenol, and the combination of both aspirin and eugenol)^17^. While ample evidence demonstrating the lipid-lowering effect of eugenol is accumulating, the mechanism by which this compound regulates lipid metabolism is still poorly understood.

Many researchers attributed the hypocholesterolemic effect of eugenol to its antioxidant properties^11,12,15^. Others suggested a direct interaction with the transient receptor potential vanilloid 1 (TRPV1) channel^18,19^. TRPV1 was identified in 1997 and is expressed in the afferent sensory neurons^20^, but has also been discovered in non-neuronal cells such as myocytes and hepatocytes^21^. TRPV1 activation was found to be essential for capsaicin-induced energy expenditure, decreasing blood cholesterol level and treating hyperlipidemia^21,22^. We hypothesized that the hypocholesterolemic effect of eugenol may be associated with the modulation of TRPV1 receptor. The rationale underlying this hypothesis is that eugenol, similar to capsaicin, contains a vanilloyl moiety and may act as an agonist activating TRPV1 receptor (Supplementary Fig. 1). The structural similarity of capsaicin and eugenol suggests that these two ligands may share the same molecular mechanism to produce their effects^18^. Accordingly, this study aims to investigate the role of TRPV1 receptor in the hypocholesterolemic and anti-steatotic effects of eugenol in a rat model. Further, we provide the eugenol-TRPV1 possible interactions at the molecular level using computer modeling and docking technique.

## Materials and Methods

### Materials

Cholesterol (purity 94%, Sigma-Aldrich, Japan), cholic acid (purity >98%, Sigma-Aldrich, New Zealand), and eugenol (purity 99%, Sigma-Aldrich, Germany) were purchased from Sigma-Aldrich in a high-purity grade. Atorvastatin calcium was kindly provided by SANA Pharmaceuticals, Amman, Jordan. The enzymatic kits for quantitative assay of TC, TG, HDL, AST, ALT, ALP and LDH, were purchased from Biosystem, Barcelona, Spain. Commercial assay kits used to determine the activity of catalase enzyme (CAT) (Cayman’s Catalase Assay Kit, Item No. 707002) and superoxide dismutase enzyme (SOD) (Cayman’s Superoxide Dismutase Assay Kit, Item No. 706002) were purchased from Cayman Chemical Company, Ann Arbor, U.S.A. Primary rabbit polyoclonal anti-TRPV1 antibody was bought from Novus Biologicals (Cat no. NB100-1617; Novus Biologicals, Littleton, CO 80120 USA). Ventana ultraView universal DAB detection kit (Cat no. 760-500), Ventana Liquid Coverslip LCS (Cat no. 650-010), Ventana Reaction Buffer (Cat no. 950-300), Ventana EZ Prep solution (Cat no. 950-102), Ventana cell conditioning solution (CC1) (Cat no. 950-124), Ventana Amplification kit (Cat no. 760-080), hematoxylin II (Cat no. 790-2208), Bluing reagent (Cat no. 760-2037) were purchased from Ventana Medical Systems Inc., Tucson, Arizona, USA.

## Methods

### Molecular modeling (Docking experiment)

The chemical structure of eugenol (Supplementary Fig. 1) was sketched in MarvinSketch 16.10.24, 2016, ChemAxon and saved in MDL mol file format. Subsequently, an ensemble of energetically accessible conformers was generated using OMEGA 2.5.1.4 software (Open Eye Scientific Software, Santa Fe, NM). The generated conformers are saved in SDF format. The 3D coordinates of TRPV1 (PDB codes: 5IS0, resolution; 3.43 Å) were collected from the Protein Data Bank^23^. Hydrogen atoms were added to the proteins using the DS Visualizer templates, DS visualizer 2.0; Accelrsy Inc. USA, for protein residues. Eugenol was docked into the binding site of the target receptor using fast, rigid exhaustive docking (FRED) within the OEDocking suite (OEDOCKING 3.2.0.2; OpenEye Scientific Software, Santa Fe, NM). The protein structures and ligand conformers are treated as rigid entities during docking simulations. The top scoring poses are optimized and assigned a final score using Chemgauss 4.

### High cholesterol and fat diet (HCFD) preparation

HCFD was prepared as previously described by Harb *et al*.^24^. In brief, cholesterol powder (2% w/w), cholic acid (1% w/w), fat (animal source) (20% w/w) and corn oil (2% w/w) were mixed with a crushed standard rat chow (referred to hereafter as normal diet; ND), and reconstituted with distilled water (1000 mL) and allowed to dry properly at 29 °C to prevent microbial contamination. This diet was prepared weekly and was stored at 4 °C until use to reduce oxidation. The composition of ND and HCFD (g/100 g), analyzed in the Feed Analysis Laboratory, Department of Animal Production, Faculty of Agriculture, The University of Jordan, Amman, is listed in Supplementary Table 1.

### Animals

The study was performed on adult male Wistar rats weighing 150–180 g at the Experimental Animal Laboratory of the Department of Biological Sciences, School of Science; University of Jordan. All animals were housed, fed and treated in accordance with the guidelines of Committee for the Purpose of Control and Supervision on Experiments on Animals (CPCSEA) and with guidelines and regulations of the University. All experimental protocols were approved by the Graduate Studies and Research Committee of the School of Sciences at the University of Jordan, Amman, Jordan.

### Experimental protocol

Rats were randomly divided into two major groups (group I and group II) at the beginning of the experiments. Group Ι was labeled as normal-diet group and fed normal diet (n = 8) whereas group II was labeled as hypercholesterolemic group and fed HCFD (n = 32). In order to make sure that hypercholesterolemia was successfully induced in group II, total cholesterol level was measured after 2 weeks of HCFD feeding, and only a rat with total cholesterol of >200 mg/dL^25^ was included in the experiment and this group was further randomly divided into 4 groups (n = 8/group): Control, atorvastatin (ATV), eugenol (Eug) 10, and Eug 100. Control group received corn oil as a vehicle whereas ATV group received the reference hypocholesterolemic drug atorvastatin, at a dose of 20 mg/kg b.w. Eug 10 and Eug 100 groups received eugenol at the doses of 10 and 100 mg/kg b.w., respectively.

All treatments were given once a day with a volume of 0.5 mL/animal by oral gavage for 4 weeks based on individual weekly body weight. Doses of eugenol were selected based on LD_50_ and previously published studies^6,14,16^.

### Blood sampling

Blood samples were collected at two-week intervals to monitor lipid profile. After overnight fasting, rats were lightly anaesthetized using diethyl ether and a blood sample (2 mL) was drawn from the retrorbital plexus of the eye, transferred to sterile vacuotainers with gel, allowed to clot at room temperature for one hour, centrifuged for 10 minutes at a speed of 3000 rpm. Serum was separated and stored in Eppendorf tubes at −20 °C for biochemical analyses.

### Termination of the experiment

At the end of the experimental period (6 weeks), rats were fasted overnight, weighed and sacrificed by an overdose of diethyl ether. Blood was collected (4 mL) and serum was obtained as described above and was stored at −20 °C until used for further analysis.

Livers were removed and weighed to calculate relative organ weight to body weight (g/100 g body weight). A small portion of liver was excised and fixed in 10% formalin saline for histopathological and immunohistochemical examination. The remaining parts of liver were stored at −70 °C until used for further analysis.

### Biochemical analyses

#### Estimation of lipid profile

Serum TC, TG and HDL levels were measured enzymatically using commercial assay kits according to the manufacturer’s instructions. LDL was calculated using the equations LDL = TC– (HDL + VLDL), where VLDL = TG/5. The atherogenic index (AI), a specific indicator for coronary heart disease, was calculated for the last week of the experiment as follows:

$${\rm{AI}}=({\rm{TC}}-{\rm{HDL}})/{\rm{HDL}}$$

#### Liver functions test

Activities of AST, ALT, ALP and LDH were measured enzymatically in the serum sample for the last week of the experiment using commercial assay kits according to the manufacturer’s instructions.

#### Antioxidant enzymes activity

Serum SOD and CAT activity was measured on enzyme linked immunosorbent assay (ELISA) plate reader at the wavelengths of 450 nm and 540 nm respectively, using a colorimetric assay kit according to the manufacturer’s instructions.

#### Estimation of hepatic HMG-CoA reductase activity

The activity of HMG-CoA reductase was measured in liver homogenate using the procedure of Rao and Ramakrishnan^26^. The ratio of HMG-CoA to mevalonate was taken as an index of enzyme activity which catalyzes the conversion of HMG-CoA to mevalonate. Lower ratios indicate higher enzyme activity and *vice versa*.

### Histopathological evaluation of hepatic steatosis and inflammation

Livers were removed from sacrificed rats and fixed in 10% formalin saline. Fixed tissues were processed for paraffin embedding. Sections, 5 µm-thick, were stained with hematoxylin–eosin. Ten random light microscopic fields of each section were examined and scored for the degree of steatosis in a blind manner under a compound light microscope at 200X (Olympus, Japan). Macrovesicular steatosis was evaluated using the grading and scoring system of Kawasaki *et al*.^27^. Quantitative evaluation of macrovesicular steatosis was reported as percentage of hepatocytes containing fat droplets equal to or larger than the size of the nucleus, often displacing the nucleus^28^.

Histological quantification of focal inflammatory cell infiltration in liver tissue was performed in a blind fashion for 5 different fields of a section at 100X magnification, and the mean of the number of focal inflammatory cell infiltration was calculated.

### Immunohistochemical detection of TRPV1

Formalin-fixed, paraffin-embedded liver tissue was cut at 4 mm, mounted on positively charged slides and dried in an oven at 85 °C for 60 min. Immunohistochemical staining was performed on the Ventana Medical System Automated immunohistochemistry (IHC) stainer (Cat no. 750–861, Tucson, Arizona, USA) using the Ventana ultraview universal 3,3’-diaminobenzidine (DAB) detection kit at The Specialty Hospital, Amman, Jordan. Each step of the immunostaining procedure was optimized and adjusted automatically with Benchmark IHC/ISH staining module (Ventana Medical Systems). Ventana liquid coverslip was used as a barrier between aqueous reagents and air to prevent evaporation throughout the automated protocol. Following deparaffinization with Ventana EZ Prep solution, antigen retrieval was performed using Ventana Tris-based buffer solution (cell conditioning solution, CC1) at 95–100 °C for 180 min. Endogenous peroxidase was blocked with ultraview DAB inhibitor (3% H_2_O_2_). The slides were rinsed with Ventana reaction buffer between steps. After rinsing, the slides were incubated at 37 °C for 20 min with a primary rabbit polyoclonal anti-TRPV1 antibody (1:300 dilution) which was manually added. Signal intensity of weak staining was increased using the Ventana amplification kit (amplifier A and B) for two incubations. Slides were then incubated with ultraview universal horseradish peroxidase (HRP) multimer (<50 ug/mL). Visualization was achieved using ultraview universal DAB chromogen (0.2% 3,3’-diaminobenzidine “DAB”) with ultraview universal DAB H_2_O_2_ (0.04% H_2_O_2_), followed by enhancement with ultraview universal DAB copper (CuSO_4_ 5 g/L). Slides were then counterstained for 6 min with hematoxylin and bluing reagent (0.1 M Li_2_CO_3_, 0.5 M Na_2_CO_3_). After rinsing, slides were removed from the instrument, manually dehydrated and coverslipped. A negative control was used to rule out nonspecific staining. For each section, four random fields were captured at 100x magnification using Leica EC3 digital camera connected to the light microscope (Leica Microsystems, Germany) and Leica application suite LAS EZ software version 1.8.0 (Leica Microsystems, Switzerland). The staining intensity of each IHC image was analyzed using Image J software with IHC Profiler plugin (National Institutes of Health, USA) and the optical density score of TRPV1 was calculated as follows:

IHC optical density score = (percentage contribution of high positive × 4 + percentage contribution of positive × 3 + percentage contribution of low positive × 2 + percentage contribution of negative × 1) /100^29^.

### Statistical analysis

Data were presented as means ± SEM, and were analyzed using one-way analysis of variance (ANOVA) followed by post hoc Fisher’s least significant difference test. Differences were considered significant when *p* < 0.05. The experimental data were analyzed using Graph Pad Prism software version (8.0.1).

### Ethical approval

The guidelines of Committee for the Purpose of Control and Supervision on Experiments on Animals (CPCSEA) were observed. All applicable guidelines for the care and use of animals at The University of Jordan were followed. All experimental protocols were approved by the Graduate Studies and Research Committee of the School of Sciences at the University of Jordan, Amman, Jordan.

## Results

### Eugenol-TRPV1 interaction

**Figure 1:**
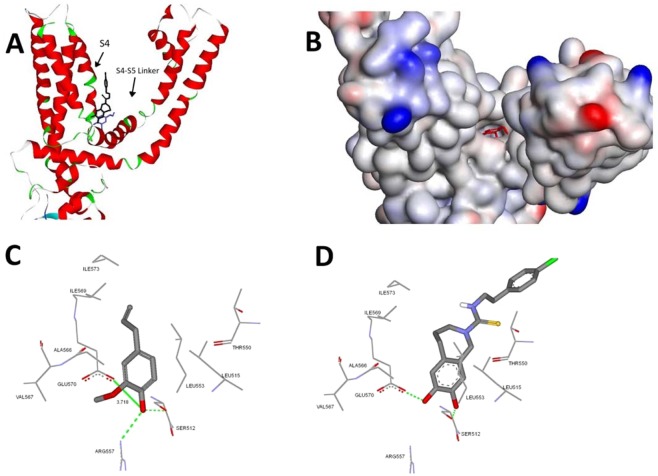
Structural model of eugenol-TRPV1 complex. (**A**) Ribbon diagram depicting relative locations of S4, S4–S5 linker and helices in the presence of capsazepine (black) and eugenol (blue). (**B**) The solvent accessible surface area of the docked eugenol within the binding sites of TRPV1 (PDB code: 5IS0). Detailed view of the docked pose of eugenol (**C**) showing the corresponding interacting amino acids within the binding sites of TRPV1 in comparison to capsazepine (**D**) co-crystallized ligand. Hydrogen bonds are indicated with green line (Number indicates distance in Å).

Figure 1A,B shows that eugenol could fit within the binding site of TRPV1 and the predicted binding mode takes a “tail-up/head-down” configuration in which the aliphatic tail points upwards while the vanilloyl group points downward to the S4–S5 linker.

Figure 1 shows several intermolecular interactions between eugenol and TRPV1, with the most relevant interaction between the ligand and the channel involves the formation of a hydrogen bond between Glu570 in the S4–S5 linker and Arg557 at the bottom of S4 by the vanilloyl head group (Fig. 1C). In contrast, capsazepine, a TRPV1 antagonist, does not form a hydrogen bond with these two amino acids (Fig. 1D). Moreover, a strong hydrogen bonding with Ser512 is also found in eugenol-TRPV1 complex. Also, several potential hydrophobic interactions were found between the hydrocarbon backbone of eugenol and Leu553, Ala566, Ile569 and Ile573 (Fig. 1C). The predicted binding energies according to Chemgauss 4 scoring function for capsazepine and eugenol were comparable (−8.415 kCal/Mol and −7.933 kCal/Mol, respectively).

### Effect of eugenol on lipid profile and AI of hypercholesterolemic rats

**Figure 2:**
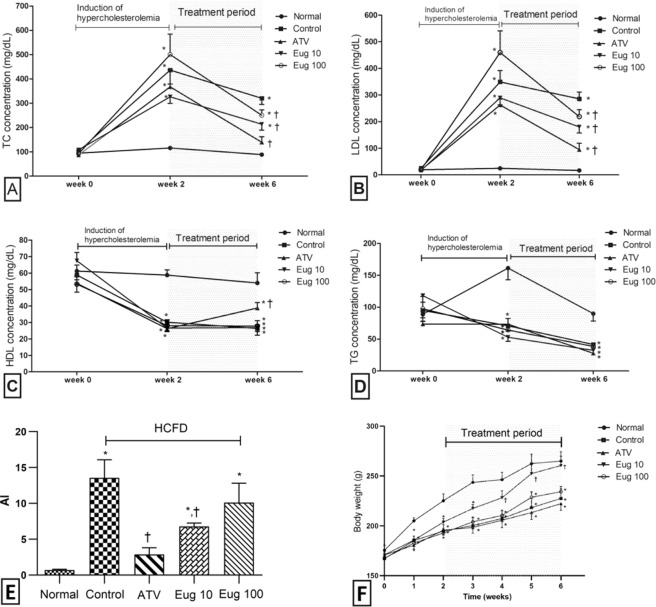
Effect of eugenol on serum lipid profile expressed in mg/dL(**A–D**), atherogenic index (**E**) and body weight (**F**) of rats. (**A)**, TC; (**B)**, LDL; (**C)**, HDL; (**D)**, TG. Normal group (●), normal diet + corn oil (vehicle); Control group (■), HCFD + corn oil; ATV group (▲), HCFD + 20 mg/kg b.w. of atorvastatin; Eug 10 group (▼), HCFD + 10 mg/kg b.w. of eugenol; Eug 100 group (○), HCFD + 100 mg/kg b.w. of eugenol. Data are expressed as means ± S.E.M. and are analyzed by one-way ANOVA followed by Fisher’s LSD test. *Significant when compared to the normal diet group; ^†^significant when compared to the control group for the same week for the same parameter.

Figure 2 illustrates the effect of two different doses of eugenol (10 and 100 mg/kg) on serum lipid profile of hypercholesterolemic rats. TC and LDL were statistically similar (*p* > 0.05) in all experimental groups at the start of the experiment (week 0). After two weeks, all the groups of animals that received an HCFD showed significant (*p* < 0.001) increase in their TC and LDL when compared to the group that received the normal diet. Among the hypercholesterolemic 4 groups, there were no significant differences (*p* > 0.05) between the control group, ATV, Eug 10, and Eug 100 groups. By the end of week 6, a significant decrease in both TC and LDL levels was observed in the ATV, Eug 10, and Eug 100 groups (*p* < 0.0001, *p* < 0.01 and *p* < 0.05, respectively) compared to the control group (Fig. 2A,B). In Fig. 2C,D, the levels of HDL and TG were statistically similar (*p* > 0.05) in all experimental groups at the start of the experiment. During the period of treatment (week 2 to week 6), the level of serum HDL and TG was statistically similar (*p* > 0.05) for the eugenol-treated groups (Eug 10 and Eug 100) and the control group.

Figure 2E shows that AI was significantly less in the ATV and Eug 10 groups (*p* < 0.001 and *p* < 0.05, respectively) compared to the control group. In contrast, AI in the control, Eug 10, and Eug 100 groups was significantly higher (*p* < 0.0001, *p* < 0.05 and *p* < 0.01, respectively) than that of the normal diet group.

### Effect of eugenol on the body weight

Figure 2F shows the body weight changes in all animal groups over the period of 6 weeks. Although all animal groups started with approximately a similar body weight, the control, ATV, Eug 100 groups did not gain weight at the same rate as those fed the normal diet (*p* < 0.001). Animals of Eug 10 group showed a significant increase in weight (*p* < 0.01) over those of the control group during the treatment period (weeks 4–6). Although the weight of this group was less than that of the normal diet group early in the experiment, they recovered and picked up weight gain so as not to be significantly different from those of the normal-diet group during the last three weeks of the experiment (weeks 4–6).

### Effects of eugenol on liver weight, structure, function, inflammation and activity of hepatic HMG-CoA reductase

**Figure 3:**
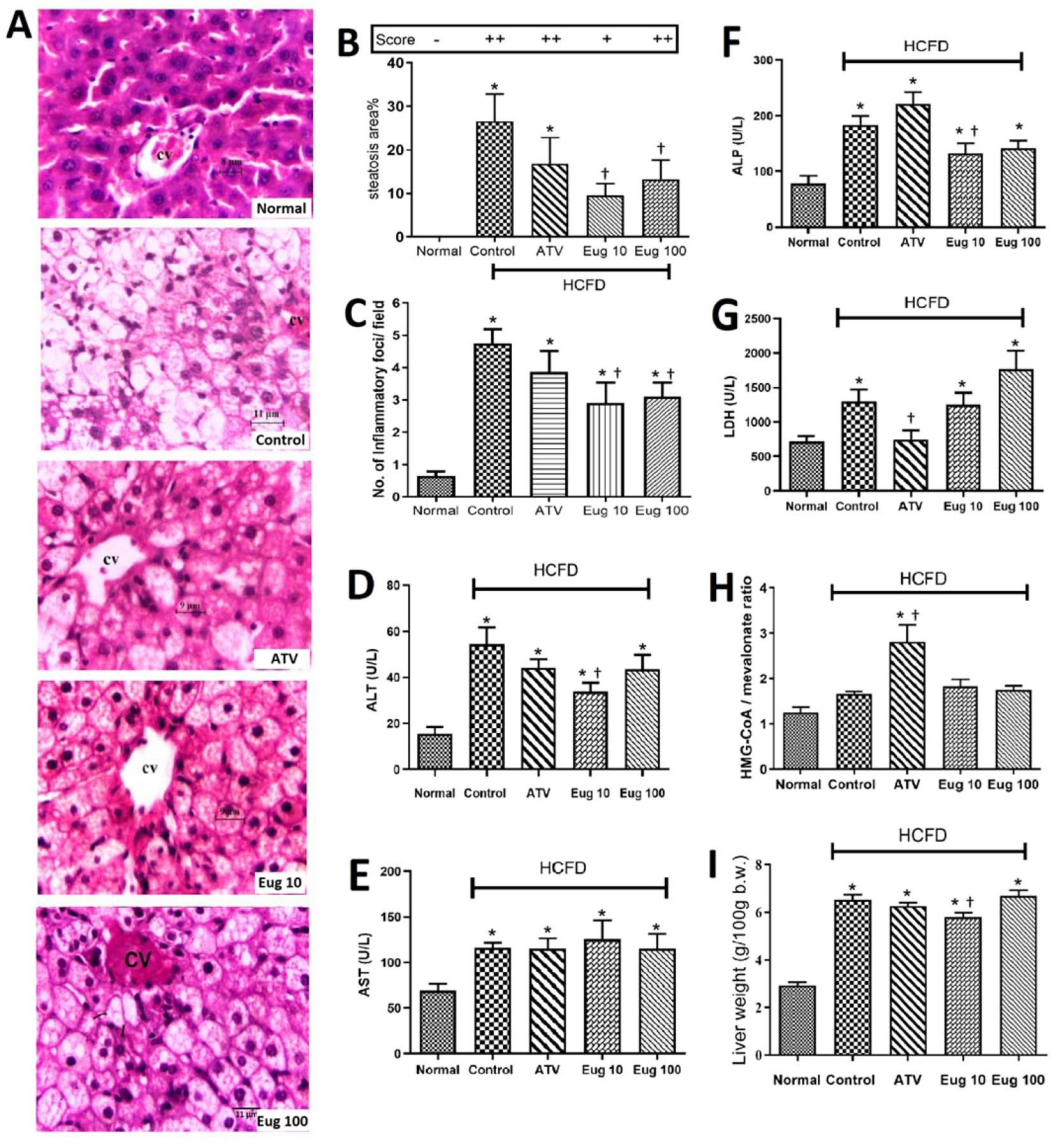
Effects of eugenol on histology of rat liver, steatosis (score and percentage), hepatic inflammation, hepatic marker enzymes, ratio of HMG Co-A/mevalonate and liver weight by the end of the 6th week (after eugenol administration for four weeks). Liver sections from rats of different groups were stained with H&E, ×400 (**A)**, steatosis score and percentage (**B**), no. of inflammatory foci/field (**C**), hepatic marker enzymes: ALT (**D**), AST (**E**), ALP (**F**), and LDH (**G**), ratio of HMG Co-A/mevalonate (**H**), and liver weight (g/100 g b.w.) (I). Normal group, normal diet + corn oil (vehicle); Control group, HCFD + corn oil; ATV group, HCFD + 20 mg/kg b.w. of atorvastatin; Eug 10 group, HCFD + 10 mg/kg b.w. of eugenol; Eug 100 group, HCFD + 100 mg/kg b.w. of eugenol. Fatty changes comprising tiny and large vacuoles, pleomorphic nuclei and swelling hepatocytes are abundant in the control group. Partial recovery in the ATV, Eug 10, and Eug 100 groups are observed. CV, central vein. Data are expressed as means ± S.E.M. and are analyzed by one-way ANOVA followed by Fisher’s LSD test. *Significant when compared to the normal diet group; ^†^significant when compared to the control group for the same parameter.

In Fig. 3A, the two eugenol-treated groups (Eug 10 and Eug 100) showed less lipid deposition and hepatocytes enlargement than the control group. Figure 3B shows that the percentage of macrovesicular steatosis decreased in Eug 10 and Eug 100 groups (*p* < 0.01 and *p* < 0.05, respectively) in comparison with the control group. Inflammatory cell infiltration also significantly decreased in both groups (Fig. 3C) as compared to the control group (*p* < 0.05).

Figure 3D,F shows decreased activity of ALT and ALP (*p* < 0.05) in Eug 10 group, but there were no detectable changes (*p* > 0.05) in the activity of AST and LDH in this group when compared to the control group (Fig. 3E,G). The higher dose of eugenol (100 mg/kg) caused no significant changes (*p* > 0.05) in all of the tested enzymes in comparison to the control group.

In Fig. 3H, there was no significant difference (*p* > 0.05) in the ratio of HMG-CoA/mevalonate between eugenol-treated groups (Eug 10 and Eug 100) and those of the control and normal diet groups. The ratio of HMG-CoA/mevalonate was significantly higher in ATV group than that of the control group (*p* < 0.001).

Liver weight was significantly increased (*p* < 0.0001) in all groups fed HCFD in comparison with the normal diet group (Fig. 3I). In Eug 10 group, liver weight was less by 10.9% (*p* < 0.05) compared to the control group.

### Effect of eugenol on the activity of serum antioxidant enzymes

**Figure 4:**
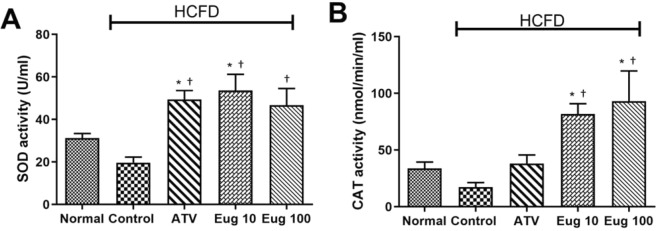
Effects of eugenol on the activity of serum SOD (**A**) and CAT (**B**) by the end of the 6^th^ week. Normal group, normal diet + corn oil (vehicle); Control group, HCFD + corn oil; ATV group, HCFD + 20 mg/kg b.w. of atorvastatin; Eug 10 group, HCFD + 10 mg/kg b.w. of eugenol; Eug 100 group, HCFD + 100 mg/kg b.w. of eugenol. Data are expressed as means ± S.E.M. and are analyzed by one-way ANOVA followed by Fisher’s LSD test. *Significant when compared to the normal diet group; ^†^significant when compared to the control group for the same parameter.

Figure 4A,B shows that eugenol-treated groups (Eug 10 and Eug 100) had a remarkable increase in the activity of both enzymes; SOD (*p* < 0.0001 and *p* < 0.01, res*p*ectively) and CAT (*p* < 0.0001) when compared to the control group. The activity of SOD was increased in ATV group as compared to the control group (*p* < 0.001).

### Effect of eugenol on the expression of TRPV1 in the liver of hypercholesterolemic rat

**Figure 5:**
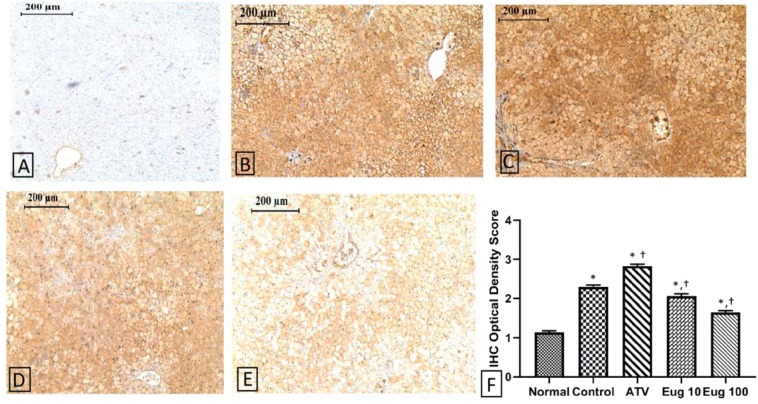
Effect of eugenol on the expression of TRPV1 in rat liver. Representative immunohistochemistry images for TRPV1 at 100X. (**A**) Normal group, normal diet + corn oil (vehicle); (**B**) Control group, HCFD + corn oil; (**C**) ATV group, HCFD + 20 mg/kg b.w. of atorvastatin; (**D**) Eug 10 group, HCFD + 10 mg/kg b.w. of eugenol; (**E**) Eug 100 group, HCFD + 100 mg/kg b.w. of eugenol. (**F**) IHC optical density score of TRPV1. Data are expressed as means ± S.E.M. and are analyzed by one-way ANOVA followed by Fisher’s LSD test. *Significant when compared to the normal diet group; ^†^significant when compared to the control group. Expression of TRPV1 was negative in (**A**) highly positive in (**C**), positive in (**B**,**D**), and low positive in (**E**).

Figure 5 shows the immunohistochemistry for TRPV1 in rat liver. A positive immunohistochemical staining of TRPV1 was detected in both membrane and cytoplasm of hepatocytes of all groups fed HCFD as compared to that of the normal-diet group (Fig. 5A–E). The IHC optical density score of TRPV1 was significantly increased (p < 0.0001) in the control, ATV, Eug 10, and Eug 100 groups in comparison to that of the normal diet group (Fig. 5F). In comparison with the control group, the IHC optical density score of TRPV1 was significantly less in Eug 10 and Eug 100 groups (p < 0.001 and p < 0.0001, respectively), whereas it was significantly higher in ATV group (p < 0.0001).

## Discussion

The present experiments confirmed the hypocholesterolemic activity of eugenol. It reduced serum total cholesterol when orally administered to hypercholesterolemic rats on a daily basis for a period of 4 weeks. Both doses of eugenol (10 mg/kg and 100 mg/kg) caused a significant decrease in the serum TC (33.4% and 21.9%, respectively) and LDL (36.9% and 23.5%, respectively) although this decrease was not as high as that induced by the reference hypocholesterolemic drug atorvastatin (56.6% for TC and 66.8% for LDL). However, neither HDL nor TG was affected by eugenol in the present experiments, suggesting that the cholesterol-lowering effect induced by eugenol was in fact exclusively attributed to a decline in LDL. More importantly, it reduced AI significantly in hypercholesterolemic rats as compared to the control group. Similar findings have been reported where eugenol was used in doses of 5 mg/kg, 18 mg/kg, and 250 mg/kg, doses which are around the range of the doses used in the present study^14,16,17^. In fact, some of these studies confirmed our findings that eugenol reduces TC and LDL but did not affect HDL level^16,17^.

Eugenol has a hepatoprotective effect in our study. We observed that liver weight in the animal group treated with 10 mg/kg of eugenol was reduced compared to that of the control group. Furthermore, histological examination showed that lipid accumulation in hepatic cells has decreased in eugenol-treated groups (10 and 100 mg/kg) by 64.4% and 50.4%, respectively, suggesting that eugenol prevented liver injury caused by hypercholesterolemia, and thus ameliorating the fatty liver. This observation is supported by the finding that eugenol has anti-fatty liver activity and represents a promising dietary supplement to treat hepatic steatosis and early stages of liver fibrosis^5^. In line with this, we showed that administration of eugenol (10 mg/kg) improved liver marker enzymes measured in serum. Eugenol, in the present study, also caused a significant decrease in ALT, a better bio-indicator of liver injury than AST^30^. It also decreased ALP, but there was no effect on either AST or LDH. These observations are in agreement with other studies which reported that eugenol reduced ALT and ALP in thioacetamide-induced hepatotoxicity^6^, streptozotocin-induced diabetes^10^, triton-induced hypercholesterolemia^14^, and high fat diet-induced fatty liver disease^5^. Hepatic inflammation was reduced by the two doses of eugenol used in this study and this observation is supported by other studies that showed eugenol has an anti-inflammatory activity^1,6^.

Eugenol possesses a potent antioxidant activity as documented by several reports^1,6,7,14,16^. In consistence with these reports, our data demonstrated that administration of eugenol at two doses (10 and 100 mg/kg) increased the activity of serum SOD and CAT.

In order to clarify the mechanism of the hypocholesterolemic effect of eugenol, we evaluated the hepatic HMG-CoA reductase activity, the rate limiting enzyme in cholesterol biosynthesis pathway. We could not detect any significant differences amongst the eugenol-treated groups and the control group. Hence, it seems that the hypocholesterolemic effect exerted by eugenol is not mediated by inhibition of cholesterol synthesis. Therefore, we evaluated the potential role of TRPV1 receptor in this hypocholesterolemic effect of eugenol. TRPV1 channel is a non-selective cationic channel which is highly permeable for Ca^2+^ and seems to play a major role in treating hyperlipidemia^21,22,31^. Our data showed that expression of TRPV1 is upregulated in the liver of hypercholesterolemic rats as demonstrated by intense staining (Fig. 5B). This observation is supported by other studies that showed TRPV1 to be upregulated in inflammatory and lipidemic conditions^31–33^. It has been reported that lipid mediators, such as oxLDL, increase the activity of TRPV1 in bone marrow-derived macrophages as evidenced by an increase in [Ca^2+^]_i_ level, and induce foam cell formation as evidenced by increased cellular levels of cholesterol and triglycerides^32^. Indeed, activation of TRPV1 signaling promotes fat accumulation, while inhibition of this signaling protects against fat accumulation^31^. Interestingly, in the present study, eugenol down regulated TRPV1 in a dose-dependent manner as demonstrated by dose-dependent decrease in immunostaining (Fig. 5D,E). The precise molecular mechanism by which eugenol influences TRPV1 is unclear and may be multifaceted. We hypothesized that chronic consumption of eugenol protected against hypercholesterolemia and fatty liver disease by causing an initial activation as an agonist, followed by long‐term desensitization of TRPV1, suggesting that the pharmacological effect of eugenol may be mediated by inhibition of TRPV1^31^. This conclusion was further bolstered by other experiments that showed that prolonged exposure of TRPV1 to agonists like capsaicin decreased the total amount of receptors in a dose- and time-dependent manner by receptor endocytosis and down-regulation through lysosomal degradation. This process is Ca^2+^ dependent and appears to be mediated by an endocytotic mechanism that is independent of clathrin and can be modulated by a protein kinase A dependent phosphorylation of serine 116^33^. Furthermore, eugenol inhibits lipid accumulation in the hepatocytes of fatty liver in mice through down regulation of gene expression of sterol regulatory element binding protein1 (SREBP1), a membrane-bound transcription factor that regulates the *de novo* lipogenesis in the liver. This process is a calcium-dependent pathway involving phosphorylation of AMP-activated protein kinase by Ca^2+^-calmodulin dependent protein kinase kinase^5^. Unexpectedly, our data revealed that atorvastatin increased the expression of TRPV1. Parallel to these findings, another member of the statin family, simvastatin, has been shown to activate TRPV1, resulting in the activation of endothelial nitric oxide synthase and angiogenesis^34^. Although atorvastatin (HMG-CoA reductase inhibitor) successfully inhibited the endogenous cholesterol biosynthesis in our study, it did not ameliorate hepatic steatosis. This implies that lipogenesis in the liver is a multifaceted process involving many genes and enzymes.

In order to explore TRPV1 as a plausible target for eugenol hypocholesterolemic effect, docking simulations were used to probe the eugenol-TRPV1 interactions employing FRED OpenEye software. The obtained results revealed that the natural compound could fit within the active site of TRPV1 and the predicted binding mode takes a “tail-up/head down” configuration in which the aliphatic tail points upwards while the vanilloyl group points downward to the S4–S5 linker.

At the molecular level, the obtained docking results revealed several important interactions between eugenol and the vanilloid receptor 1. The most relevant interaction between the ligand and the channel involves the formation of a hydrogen bond between Glu570 in the S4–S5 linker and Arg557 at the bottom of S4 by the vanilloyl head group, an interaction that contributes to make the linker and the S4 domains a single rigid unit. Interestingly, the structure of TRPV1 in complex with capsaicin, a well-known modulator of TRPV1, revealed similar interaction^35^, while capsazepine head group does not form the hydrogen bond between these two amino acids. Moreover, a strong hydrogen bonding with Ser512 is also found. These multiple polar interactions enhance the stability of eugenol-TRPV1 complex. Finally, several potential hydrophobic interactions were found between the hydrocarbon backbone of eugenol and Leu553, Ala566, Ile569 and Ile573. It is noteworthy that the amino acid Arg557 was recently reported to form hydrogen bonding with cholesterol of the inner leaflet of a lipid bilayer with a comparable binding energy to that observed with eugenol in the present study. This H-bond is only formed with the closed conformation of the TRPV1 channel^36^, suggesting that the binding of eugenol to Arg557 may actually regulate the channel function. Importantly, the obtained *in silico* results in this work is in agreement with the hypothesis reported in several previous computational studies^35,37,38^ that TRPV1 modulators act as “molecular glue” between the S4–S5 linker and the S1–S4 domain. Although many small molecules could fit inside the binding pocket, there are other important aspects which give confidence in the computational results. These include: 1. The correct orientation (optimum binding pose), 2. The key amino acids involved in the interactions within the active site, 3.Type of interactions and strength of binding (e.g., distance-based) and all these were achieved with eugenol-TRPV1 binding, thus we can strongly indicate that these molecular docking results are specific and could show potential binding within the active site of TRPV1.

A notable reduction was observed in the rate of increase of body weight of hypercholesterolemic rats relative to the normal rats and this could be due to anorexia which may lead to reduction in food intake^39^, and/or due to low carbohydrate and high fat in HCFD relative to that in ND. It has been reported that low carbohydrate and high fat diet which is known as “ketogenic diet” causes hepatic insulin resistance, and induces type 2 diabetes and non-alcoholic fatty liver disease^40^. Our data show that administration of eugenol (10 mg/kg b.w.) to rats on HCFD could significantly restore body weight pattern to near normal during the treatment period (weeks 4–6). This improvement in body weight gain may be associated with the improvement of lipid profile, liver function, insulin level, antioxidant system and reduction of the severity of fatty liver disease, which collectively lead to better metabolism and growth. A recent study has shown that intragastric administration of 10 mg/kg of eugenol to diabetic rats for 30 days caused a significant decline in the levels of blood glucose, increased plasma insulin level, and improved body weight and hepatic glycogen content^10^.

In general, this study shows that the hypocholesterolemic effect exerted by eugenol was not dose dependent since dose 10 mg/kg was more effective than dose 100 mg/kg of eugenol. A possible reason may be the toxicity associated with higher dosage of eugenol. Eugenol at a dose of 100 mg/kg caused insignificant splenomegaly (data not shown) which may be associated with immunotoxicity^41^. This dose-independent activity of eugenol could be attributed to the term known as “hormesis”, a term introduced by Goldman^42^ meaning “the beneficial effect of a low level exposure to an agent that is harmful at high levels”.

## Conclusion

Eugenol plays a vital role in reducing LDL, protecting liver from severe steatosis, reducing inflammation, and improving antioxidant status. As reported by other laboratories, this study confirms the hypolipidemic and anti-fatty liver effects of eugenol. Data and molecular docking suggest that these effects are mediated, at least in part, by TRPV1.

## Supplementary information

Supplementar Figure 1

Supplementary table 1

## References

[1] Kaur G, Athar M, Alam MS (2010). Eugenol precludes cutaneous chemical carcinogenesis in mouse by preventing oxidative stress and inflammation and by inducing apoptosis. Mol. Carcinogen..

[2] Lahlou S, Interaminense LFL, Magalhães PJC, Leal-Cardoso JH, Duarte GP (2004). Cardiovascular effects of eugenol, a phenolic compound present in many plant essential oils, in normotensive rats. J. Cardiovasc. Pharmacol..

[3] Additives JFWECof. WHO technical report series 934: Evaluation of certain food additives. 49–54 (World Health Organization: Geneva, 2006). 17069402

[4] Nejad SM, Özgunes H, Basaran N (2017). Pharmacological and toxicological properties of eugenol. Turk. J. Pharm. Sci..

[5] Jo HK, Kim GW, Jeong KJ, Kim DY, Chung SH (2014). Eugenol ameliorates hepatic steatosis and fibrosis by down-regulating SREBP1 gene expression via AMPK-mTOR-p70S6K signaling pathway. Biol. Pharm. Bull..

[6] Yogalakshmi B, Viswanathan P, Anuradha CV (2010). Investigation of antioxidant, anti-inflammatory and DNA-protective properties of eugenol in thioacetamide-induced liver injury in rats. Toxicology.

[7] Khunkitti W, Veerapan P (2012). Hahnvajanawong, C. *In vitro* bioactivities of clove buds oil (*Eugenia caryophyllata*) and its effect on dermal fibroblast. Int. J. Pharm. Pharm. Sci..

[8] Al-Trad B, Alkhateeb H, Alsmadi W, Al-Zoubi M (2019). Eugenol ameliorates insulin resistance, oxidative stress and inflammation in high fat-diet/streptozotocin-induced diabetic rat. LifeSciences.

[9] Damiani CEN, Rossoni LV, Vassallo D (2003). Vasorelaxant effects of eugenol on rat thoracic aorta. Vasc. Pharmacol..

[10] Srinivasan S (2014). Ameliorating effect of eugenol on hyperglycemia by attenuating the key enzymes of glucose metabolism in streptozotocin-induced diabetic rats. Mol. Cell Biochem..

[11] Suanarunsawat T, Ayutthaya WDN, Songsak T, Thirawarapan S, Poungshompoo S (2010). Antioxidant activity and lipid-lowering effect of essential oils extracted from *Ocimum sanctum* L. leaves in rats fed with a high cholesterol diet. J. Clin. Biochem. Nutr..

[12] Karimi I (2010). Anti-hyperlipidaemic effects of an essential oil of *Melissa officinalis* L. in cholesterol-fed rabbits. J. Applied Bio. Sci..

[13] Balasasirekha R, Lakshmi UK (2012). Effect of cloves and turmeric on hyperlipidemics. J. Hum. Ecol..

[14] Venkadeswaran K (2014). Antihypercholesterolemic and antioxidative potential of an extract of the plant, *Piper betle*, and its active constituent, eugenol, in triton WR-1339-induced hypercholesterolemia in experimental rats. Evid. Based Complement. Alternat. Med..

[15] Al-Okbi SY, Mohamed DA, Hamed TE, Edris AE (2014). Protective effect of clove oil and eugenol microemulsions on fatty liver and dyslipidemia as components of metabolic syndrome. J. Med. Food.

[16] Elbahy DA, Madkour HI, Abdel-Raheem MH (2015). Evaluation of antihyperlipidemic activity of eugenol in triton induced hyperlipidemia in rats. Int. J. Res. Stud. Biosci..

[17] Karam I (2015). Regulation effect of Aspirin Eugenol Ester on blood lipids in Wistar rats with hyperlipidemia. BMC Vet. Res..

[18] Yang BH (2003). Activation of vanilloid receptor 1 (VR1) by eugenol. J. Dent. Res..

[19] Bishnoi M, Kondepudi KK, Baboota RK, Dubey R, Boparai RK (2013). Role of transient receptor potential channels in adipocyte biology. Expert Rev. Endocrinol. Metab..

[20] Caterina MJ (1997). The capsaicin receptor: a heat-activated ion channel in the pain pathway. Nature.

[21] Wang P, Liu D, Zhu Z (2011). Transient receptor potential vanilloid type-1 channel in cardiometabolic protection. J. Korean Soc. Hypertens..

[22] Wang GY, Wang LL, Xu B, Zhang JB, Jiang JF (2013). Effects of moxibustion temperature on blood cholesterol level in a mice model of acute hyperlipidemia: Role of TRPV1. Evid. Based Complement. Alternat. Med..

[23] Yuan G, Erhu C, David J, Yifan C (2016). TRPV1 structures in nanodiscs reveal mechanisms of ligand and lipid action. Nature.

[24] Harb AA, Bustanji YK, Abdalla SS (2018). Hypocholesterolemic effect of β-caryophyllene in rats fed cholesterol and fat enriched diet. J. Clin. Biochem. Nutr..

[25] Sinnott B, Syed I, Sevrukov A, Barengolts E (2006). Coronary calcification and osteoporosis in men and postmenopausal women are independent processes associated with aging. Calcif. Tissue Int..

[26] Rao AV, Ramakrishnan S (1975). Indirect assessment of hydroxymethylglutaryl-CoA reductase (NADPH) activity in liver tissue. Clin. Chem..

[27] Kawasaki T (2009). Rats fed fructose-enriched diets have characteristics of nonalcoholic hepatic steatosis. J. Nutr..

[28] Mennesson N (2009). Liver steatosis quantification using magnetic resonance imaging: A prospective comparative study with liver biopsy. J. Comput. Assist. Tomogr..

[29] Jafari SMS, Hunger RE (2017). IHC Optical Density Score: A New Practical Method for Quantitative Immunohistochemistry Image Analysis. Appl. Immunohistochem. Mol. Morphol..

[30] Williamson, E. M., Okpako, D. T. & Evans, F. J. Selection, preparation and pharmacological evaluation of plant materials. In: Williamson EM, editor. Pharmacological methods in phytotherapy research, Chichester: John Wiley, 184–186 (1996).

[31] Motter AL, Ahern GP (2008). TRPV1-null mice are protected from diet-induced obesity. FEBS letters.

[32] Zhao JF (2013). Activation of TRPV1 prevents OxLDL-induced lipid accumulation and TNF-α-induced inflammation in macrophages: role of liver X receptor α. Mediat. Inflamm..

[33] Sanz-Salvador L, Andrés-Borderia A, Ferrer-Montiel A, Planells-Cases R (2012). Agonist- and Ca^2+^-dependent desensitization of TRPV1 channel targets the receptor to lysosomes for degradation. J. Biol. Chem..

[34] Su KH (2014). The essential role of transient receptor potential vanilloid 1 in simvastatin‐induced activation of endothelial nitric oxide synthase and angiogenesis. Acta. Physiolo..

[35] Carnevale V, Rohacs T (2016). TRPV1: A Target for Rational Drug Design. Pharmaceuticals.

[36] Saha S (2017). Preferential selection of arginine at the lipid-water-interface TRPV1 during vertebrate evolution of correlates with its snorkeling behaviour and cholesterol interaction. Sci. Reports.

[37] Yang F (2015). Structural mechanism underlying capsaicin binding and activation of the TRPV1 ion channel. Nat. Chem. Biol..

[38] Darre L, Domene C (2015). Binding of capsaicin to the TRPV1 ion channel. Mol. Pharm..

[39] Ji G, Zhao X, Leng L, Liu P, Jiang Z (2011). Comparison of dietary control and atorvastatin on high fat diet induced hepatic steatosis and hyperlipidemia in rats. Lipids Health Dis..

[40] Jornayvaz FR (2010). A high-fat, ketogenic diet causes hepatic insulin resistance in mice, despite increasing energy expenditure and preventing weight gain. Am. J. Physiol. Endocrinol. Metab..

[41] Gao S, Wang Y, Zhang P, Dong Y, Li B (2008). Subacute oral exposure to dibromoacetic acid induced immunotoxicity and apoptosis in the spleen and thymus of the mice. Toxicol. Sci..

[42] Goldman M (1996). Cancer risk of low-level exposure. Science.

