# Supplementary material for: Eugenol Reduces LDL Cholesterol and Hepatic Steatosis in Hypercholesterolemic Rats by Modulating TRPV1 Receptor
Source: Sci Rep. 2019 Sep 30;9:14003. doi: 10.1038/s41598-019-50352-4 (PMC6768860; doi:10.1038/s41598-019-50352-4)
Supplement: Supplementary file 1 — Supplementar Figure 1 [file 41598_2019_50352_MOESM1_ESM.pdf]

Eugenol Reduces LDL Cholesterol and Hepatic Steatosis in Hypercholesterolemic Rats by Modulating TRPV1 Receptor

Amani A. Harb, Yasser K. Bustanji, Ihab M. Almasri, Shtaywy S. Abdalla

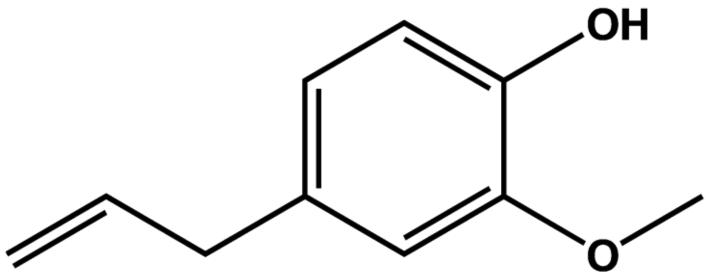

Supplementary Fig. 1: Chemical structure of eugenol
