# Supplementary material for: Eugenol Reduces LDL Cholesterol and Hepatic Steatosis in Hypercholesterolemic Rats by Modulating TRPV1 Receptor
Source: Sci Rep. 2019 Sep 30;9:14003. doi: 10.1038/s41598-019-50352-4 (PMC6768860; doi:10.1038/s41598-019-50352-4)
Supplement: Supplementary file 2 — Supplementary table 1 [file 41598_2019_50352_MOESM2_ESM.pdf]

Eugenol Reduces LDL Cholesterol and Hepatic Steatosis in Hypercholesterolemic Rats by Modulating TRPV1 Receptor

Amani A. Harb, Yasser K. Bustanji, Ihab M. Almasri, Shtaywy S. Abdalla

Supplementary Table 1: Experimental diets composition (g/100g**)**

**Component**	**ND***	**HCFD***
Crude fat	4.23	3.17
Crude protein	16.51	12.38
Total ash	6.22	4.68
Moisture	8.63	6.47
Crude fiber	6.31	4.73
Carbohydrate	58.1	43.57
Animal fat	0	20
Corn oil	0	2
Cholesterol	0	2
Cholic acid	0	1

*ND, normal diet; HCFD, high cholesterol and fat diet.
